# Atrio-aortic erosion caused by Amplatzer Atrial Septal Occluder – a case report

**DOI:** 10.1186/s13019-021-01411-3

**Published:** 2021-03-20

**Authors:** Christine E. Kamla, Joscha Buech, Philipp M. Doldi, Christian Hagl, Gerd Juchem, Alexey Dashkevich

**Affiliations:** 1 Department of Cardiac Surgery, LMU University Hospital, Marchioninistrasse 15, 81377 Munich, Germany; 2 Department of Internal Medicine I – Cardiology, LMU University Hospital, Marchioninistrasse 15, 81377 Munich, Germany

**Keywords:** Atrial septal defect, Cardiac tamponade, Septal Occluder device, Case report

## Abstract

**Background:**

In specialized centers, percutaneous closure using specific occluders is the first-choice treatment in atrial septal defects (ASD). Late complications after this intervention, such as erosion of the aorta or the atria, are rare and have not been sufficiently approached and dealt with in literature. In our clinic we have been faced with the problematic situation of diagnosing and treating such cases. That is why, we have decided to share our experience with other colleagues.

**Case presentation:**

We present two cases of severe late complications after percutaneous closure of atrial septal defects (ASD). In both cases, the atrial septal occluder (Amplatzer™ Atrial Septal Occluder Device, Abbott, Chicago USA) caused the erosion between the left atrium and the aortic root. The atrio-aortic erosion led to acute cardiac tamponade with upper venous congestion and shock. As the bleeding source remained undetectable for any imaging tools, a diagnostical sternotomy remained the only solution.

The cause of the acute bleeding was discovered to be the erosion between the left atrium and the aortic root. The treatment consisted in the removal of the occluder, direct suturing of the perforated areas and the surgical closure of the remaining ASD. The patients fully recovered within the nine to fourteen days’ hospital stay. Six months after surgery both patients were well and able to recover their daily routine.

**Conclusions:**

The atrio-aortic erosion after percutaneous closure of atrial septal defects is a surgical emergency. The more so, since it can be complicated by the absence of specific symptoms. A key-element in the diagnosis of this rare pathology remains the medical history of the patient, which the surgeon has to consider thoroughly and launch the diagnostic sternotomy without delay.

## Background

ASD represent 7–10% of all congenital heart defects [1]. Meanwhile, the percutaneous closure of the abnormal connection between the right and the left atrium using a metal device is an established alternative to open surgical approach. If done in specialized centres, the percutaneous treatment shows lower complication rates than the surgical approach [2, 3].

Nevertheless, several complications regarding the implantation of atrial septum occluders (ASO) have been reported. Sequelae which appear in the early post-interventional phase are: residual shunts, embolization of the device or thromboembolism due to incomplete endothelialisation, compression of the atrioventricular valves, V. Cava superior or the right upper pulmonary vein and emergency conversion to a surgical repair [4]. Late complications such as erosion of the Aorta or the atria, perforation and cardiac rupture or aorto-atrial fistula formation might be life-threatening [5, 6]. Nevertheless, different types of devices are associated with different levels of occurrences of these late complications. Most of the reported cases of erosion are associated to the Amplatzer Septal Occluder [7]. According to US data, erosion due to the Amplatzer Septal Occluder occurs in maximum 3 of 1000 patients [8].

We describe here a series of two patients treated in our clinic for late atrio-aortic erosions after ASO placement, who required emergent surgical correction.

## Case presentation

From December 2017 to March 2018 two patients with late complications after interventional treatment of ASD were surgically treated in our department. The two patients were men of 59 and 65 years of age.

They both had a history of percutaneous closure of ASD II using Amplatzer ASO Device 1 year, and 10 years before, due to progressive dysfunction of the right ventricle. At that point, the patients suffered from poor physical resilience and commencing end-organ venous congestion. Pulmonary hypertension had been excluded. Both interventions were performed in high specialized centers, without any complications and the patients were discharged from the hospital after a short monitoring period of 5 and 7 days, respectively. The patients recovered properly after the intervention, especially the right-heart function.

Except for high blood pressure under multiple antihypertensive treatment, no other chronic disease had been known in either patient’s medical history.

Both patients presented themselves with sudden-onset chest pain, weakness and confusion, to peripheral emergency departments.

The clinical examination revealed, alongside hypotension and tachycardia, cold and clammy extremities in both patients. One of the patients presented the complete clinical image of a pericardial tamponade including venous distension in the neck area and a paradoxical pulse.

**Fig. 1:**
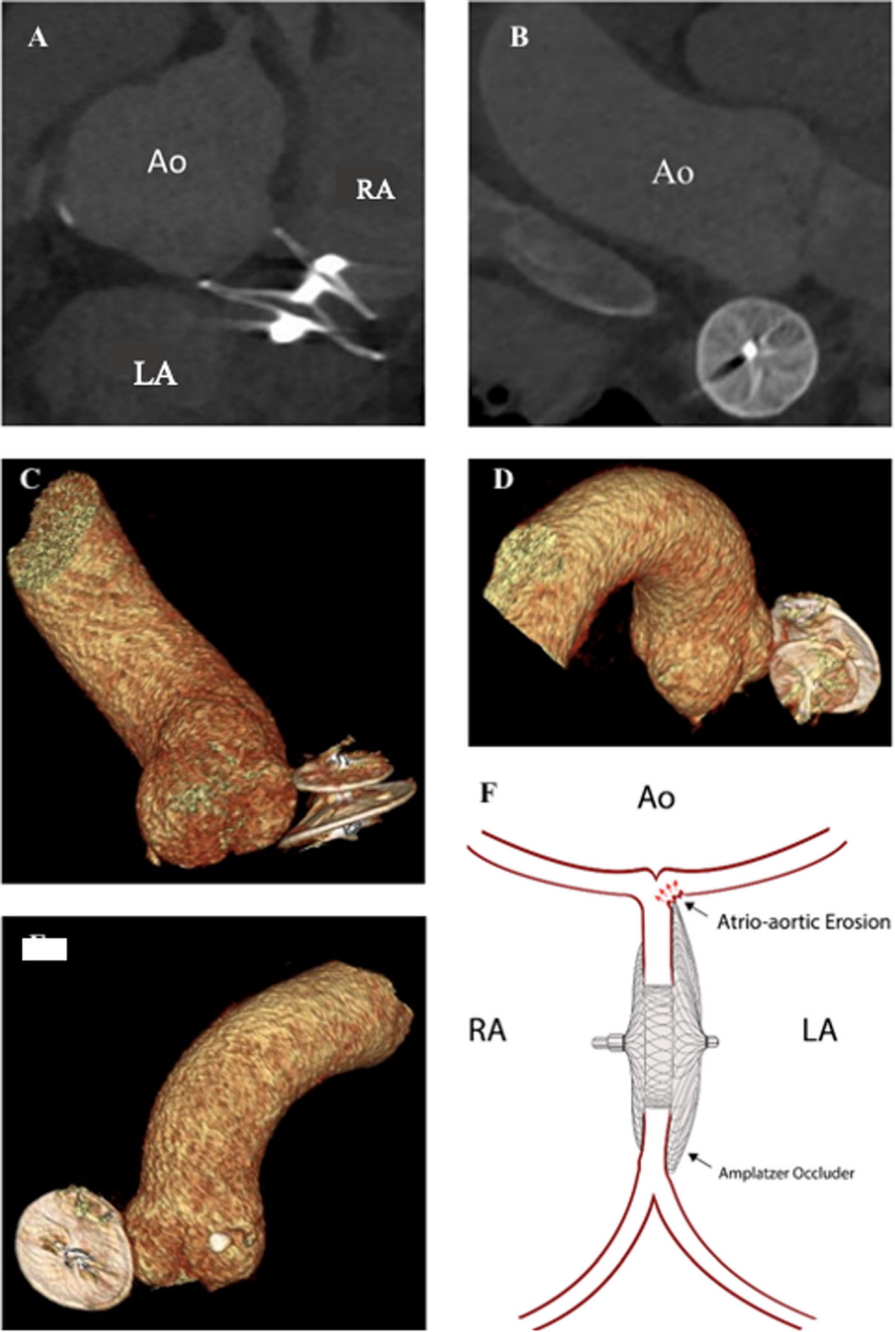
CT-angiography, 3D-reconstruction and schematic visualization of anatomic proximity of ascending aorta and Amplatzer-Device. **a** CT-angiography of Aorta ascendens and Amplatzer-device, sagittal view; **b** CT-angiography of Aorta ascendens and Amplatzer device, coronar view; **c** 3D-Reconstruction of CT- angiography; sagittal view; congruent image of **a**; **d** 3D Reconstruction of CT-Angiography; posterior view. **e** 3D Reconstruction of CT-Angiography; coronar view, congruent image of **b**. **f** Schematic visualization of atrio-aortic erosion; Ao = aorta ascendens, LA = left atrium, RA = right atrium

The revealing of cardiac tamponade with dense pericardial effusion by transthoracic echocardiography in both cases raised the suspicion for acute aortic syndrome. However, CT scans could only confirm the pericardial effusion without providing any further relevant findings (Fig. 1). Especially the bleeding source could not be visualized. Emergency sternotomy due to cardiac tamponade with hemodynamic instability remained the only option. After full sternotomy a bleeding from the bottom of the aortic root was revealed in both patients.

Total cardiopulmonary bypass was chosen for exact localization of the bleeding-source and further surgical correction. Therefore, bicaval cannulation has been performed after direct incision of both superior and inferior venae cavae.

As the ascending aorta showed no obvious pathological changes, its distal site has been used for arterial cannulation. Crystalloid cardioplegia was used for myocardial protection. The right atrium and the ascending aorta were opened to access the lesion. In both cases erosions between the left atrial roof and the noncoronary aortic sinus have been discovered as being the source of the bloody pericardial effusion. The ASO was removed and the remaining septal defects were closed using bovine pericardium. Simple running suture has been performed using polypropylene sutures.

The perforated areas of the left atrium and the noncoronary aortic sinus were closed by direct running suture.

The postoperative evolution showed no neurological or other major complications. The patients fully recovered and were discharged nine and fourteen days after surgery.

Six months after surgery both patients presented themselves for check-up in our outpatient unit. Both showed no signs of heart failure and had an almost normal physical resilience. Transthoracic echocardiography showed normal right- and left-heart function with no signs of residual shunts. Nevertheless, transoesophageal echocardiography has not been performed during follow-up for better assessment of ASD closure.

## Discussion and conclusions

Interventional ASD closure using ASO is the current therapy gold standard [8]. Although the overall safety of this approach has been stated in numerous studies and severe complications are rare, they still might be life-threatening and need urgent diagnosis and therapy. But since the diagnostic methods are not specific, severe complications like the late atrio-aortic erosion remain challenging. Due to the tight positioning of the damaged anatomical structures to the ASO, standard imaging tools cannot reveal the erosion site as the bleeding source. Figure 1 shows initial CT scans including 3D reconstructions of both patients, underlining the anatomical proximity of the involved structures.

In the acute situation, with the affected patient developing signs of shock due to pericardial tamponade of unknown origin, explorative sternotomy seems the best diagnostic and therapeutic procedure. Pericardiocentesis with subsequent angiography might be used as a simple diagnostic method. However, the atrio-aortic erosion requests an open-surgical treatment.

Possible risk factors for the late development of erosion might be hypertension and aortic aneurysm, both leading to increased tension between the aortic root, atrial wall and device rim. Based on the same pathophysiology, enlarged left atria caused by severe mitral valve insufficiency could also favor the development of atrio-aortic erosions. However, postoperative echocardiography showed normal atrial volume - left atrium: 42 ml (4 chamber view); right atrium: 46 ml - and no relevant valve insufficiency in both patients. Also, no aortic aneurysms have been described in our patients’ medical history, but both suffered from high blood pressure requiring combined antihypertensive therapy.

ASO implantation shows technical difficulties in large defects because of anatomic limitations. Implantation despite size mismatch between the ASO and the septal defect may lead to complications such as residual shunts or erosions of the cardiac structures, as discussed by Alain Fraisse et al. [9].

Before interventional closure, our patients suffered from ASD II with a size of 21 and 26 mm, respectively.

To our knowledge, only a few similar cases of atrio-aortic erosions following ASO implantation have been published [10–12].

Hans-Henning Sauer et al. described a case in a young girl 1 day after implantation of the Amplatzer device [13]. As an eventual risk factor for device erosion they identified a deficient anterosuperior rim of the septum defect. However, this causal relationship has not been proved. Loeffelbein et al. reported a case of atrial wall and aortic root penetration by an Amplatzer ASO in a boy with Marfan syndrome. In this case, as the author suggests, the altered connective tissue might play an important role in the dislocation of the device. Moreover, the occluder might alter the non-rigid tissue more easily than a healthy one [14].

Nevertheless, none of the supposed risk factors applies in the cases of our patients presented in this report. Neither a deficient septum defect rim, nor a systemic deficiency of the connective tissue had been mentioned in the medical history of our patients.

Most of the cases of hemopericardium following ASO implantation occurred within 72 h after the intervention. The numbers regarding the incidence of perforation or rupture due to ASO vary between 0.1 and 4% in the USA. In 2013, the FDA published a report of erosion events following percutaneous ASD closure and quoted that such events occur in 1–3 of every 1000 implantations [8]. Nevertheless, the published data and our clinical experience only relate to a specific type of device, namely the St. Jude Amplatzer ASO and this type of complication seems not to take place when using other devices.

To sum up, the atrio-aortic erosion after percutaneous closure of atrial septal defects is a surgical emergency complicated by the absence of specific symptoms and indices. The only reliable diagnostic method remains the diagnostic sternotomy, which should be launched without delay, taking into account the patient’s medical history.

In our opinion, of great importance in avoiding late complications after ASO implantation is the correspondence between the ASD-size and the size of the implanted device. Therefore, the precision of the measurements within the pre-implantation investigations is essential. Large ASD’s shall be treated by means of open-heart surgery, while the percutaneous closure using ASO should remain gold standard in the therapy of small ASD’s.

## Data Availability

All data generated or analysed during this study are included in this published article [and its supplementary information files].

## Abbreviations

ASD: Atrial septal defects
ASO: Atrial septal occluder
